# Polyphenols and Their Impact on the Prevention of Neurodegenerative Diseases and Development

**DOI:** 10.3390/nu15153454

**Published:** 2023-08-04

**Authors:** Izabela Grabska-Kobyłecka, Piotr Szpakowski, Aleksandra Król, Dominika Książek-Winiarek, Andrzej Kobyłecki, Andrzej Głąbiński, Dariusz Nowak

**Affiliations:** 1 Department of Clinical Physiology, Medical University of Lodz, Mazowiecka 6/8 Street, 92-215 Łódź, Poland; 2 Department of Neurology and Stroke, Medical University of Lodz, Zeromskiego 113 Street, 90-549 Łódź, Poland; piotr.szpakowski@umed.lodz.pl (P.S.); dominika.ksiazek@umed.lodz.pl (D.K.-W.); andrzej.glabinski@umed.lodz.pl (A.G.); 3 Department of Experimental Physiology, Medical University of Lodz, Mazowiecka 6/8 Street, 92-215 Łódź, Poland; aleksandra.krol@umed.lodz.pl; 4 Interventional Cardiology Lab, Copernicus Hospital, Pabianicka Str. 62, 93-513 Łódź, Poland; andkob@interia.pl

**Keywords:** polyphenols, neurodegenerative diseases, neuroprotection, Alzheimer’s disease, Parkinson’s disease, diet

## Abstract

It is well known that neurodegenerative diseases’ development and progression are accelerated due to oxidative stress and inflammation, which result in impairment of mitochondrial function, cellular damage, and dysfunction of DNA repair systems. The increased consumption of antioxidants can postpone the development of these disorders and improve the quality of patients’ lives who have already been diagnosed with neurodegenerative diseases. Prolonging life span in developed countries contributes to an increase in the incidence ratio of chronic age-related neurodegenerative disorders, such as PD (Parkinson’s disease), AD (Alzheimer’s disease), or numerous forms of age-related dementias. Dietary supplementation with neuroprotective plant-derived polyphenols might be considered an important element of healthy aging. Some polyphenols improve cognition, mood, visual functions, language, and verbal memory functions. Polyphenols bioavailability differs greatly from one compound to another and is determined by solubility, degree of polymerization, conjugation, or glycosylation resulting from chemical structure. It is still unclear which polyphenols are beneficial because their potential depends on efficient transport across the BBB (blood-brain barrier), bioavailability, and stability in the CNS (central nervous system). Polyphenols improve brain functions by having a direct impact on cells and processes in the CNS. For a direct effect, polyphenolic compounds must be able to overcome the BBB and accumulate in brain tissue. In this review, the latest achievements in studies (animal models and clinical trials) on the effect of polyphenols on brain activity and function are described. The beneficial impact of plant polyphenols on the brain may be summarized by their role in increasing brain plasticity and related cognition improvement. As reversible MAO (monoamine oxidase) inhibitors, polyphenols are mood modulators and improve neuronal self-being through an increase in dopamine, serotonin, and noradrenaline amounts in the brain tissue. After analyzing the prohealth effects of various eating patterns, it was postulated that their beneficial effects result from synergistic interactions between individual dietary components. Polyphenols act on the brain endothelial cells and improve the BBB’s integrity and reduce inflammation, thus protecting the brain from additional injury during stroke or autoimmune diseases. Polyphenolic compounds are capable of lowering blood pressure and improving cerebral blood flow. Many studies have revealed that a nutritional model based on increased consumption of antioxidants has the potential to ameliorate the cognitive impairment associated with neurodegenerative disorders. Randomized clinical trials have also shown that the improvement of cognitive functions resulting from the consumption of foods rich in flavonoids is independent of age and health conditions. For therapeutic use, sufficient quantities of polyphenols must cross the BBB and reach the brain tissue in active form. An important issue in the direct action of polyphenols on the CNS is not only their penetration through the BBB, but also their brain metabolism and localization. The bioavailability of polyphenols is low. The most usual oral administration also conflicts with bioavailability. The main factors that limit this process and have an effect on therapeutic efficacy are: selective permeability across BBB, gastrointestinal transformations, poor absorption, rapid hepatic and colonic metabolism, and systemic elimination. Thus, phenolic compounds have inadequate bioavailability for human applications to have any beneficial effects. In recent years, new strategies have been attempted in order to exert cognitive benefits and neuroprotective effects. Converting polyphenols into nanostructures is one of the theories proposed to enhance their bioavailability. The following nanoscale delivery systems can be used to encapsulate polyphenols: nanocapsules, nanospheres, micelles, cyclodextrins, solid lipid nanoparticles, and liposomes. It results in great expectations for the wide-scale and effective use of polyphenols in the prevention of neurodegenerative diseases. Thus far, only natural polyphenols have been studied as neuroprotectors. Perhaps some modification of the chemical structure of a given polyphenol may increase its neuroprotective activity and transportation through the BBB. However, numerous questions should be answered before developing neuroprotective medications based on plant polyphenols.

## 1. Introduction

Selective neuronal vulnerability to various stressors, including oxidative stress, is a hallmark of the pathophysiology of neurodegenerative diseases [1]. The involvement of oxidative stress and inflammation in the development and progression of neurodegenerative disorders is well documented in epidemiological studies [2,3]. Due to the high content of polyunsaturated fatty acids, high oxygen demand, and a relatively low level of antioxidant compounds, the brain tissue is extremely sensitive to oxidative damage [4]. Moreover, increasing the accumulation of reactive oxygen species (ROS) in the brain expands the permeability of the blood-brain barrier (BBB), leading to neuroinflammation and degeneration of neurons [5]. Impairment of mitochondrial function, cellular damage, and dysfunction of DNA repair systems caused by increased oxidative stress are factors that critically enhance the progression of neurodegenerative disorders [6]. Thus, in recent years, the prevention of oxidative damage by increasing the consumption of antioxidants has been taken into account as a possibility that improves the quality of life of patients with neurodegenerative diseases. Nevertheless, for the clinical use of antioxidants, it is necessary to carefully select the substances with the greatest therapeutic benefits, determining their exact mechanism of action and bioavailability. Moreover, the current state of knowledge indicates that most antioxidant compounds are characterized by low penetration by the BBB. Therefore, it is extremely important to search for options that increase their efficiency and availability. Well-known substances eliciting strong antioxidant properties are polyphenols—natural compounds synthesized exclusively by plants with chemical features related to phenolic substances. Thus, they have potential health-promoting effects. These molecules or classes of natural substances are characterized by at least two phenyl rings and one or more hydroxyl substituents. Polyphenols can be simply classified into flavonoids and nonflavonoids or be subdivided into many subclasses depending on the number of phenol units within their molecular structure, substituent groups, and/or the linkage type between phenol units. Polyphenols are widely distributed in plant tissues, where they mainly exist in the form of glycosides or aglycones. The structural diversity of flavonoid molecules arises from variations in hydroxylation pattern and oxidation state, resulting in a wide range of compounds: flavanols, anthocyanidins, anthocyanins, isoflavones, flavones, flavonols, flavanones, and flavanonols. Nonflavonoid molecules are phenolic acids, hydroxycinnamic acids, lignans, stilbenes, and tannins. The neurobiological activity of plant polyphenols also depends on their availability in brain tissue. Actually, it is clear that some polyphenols from the diet are able to cross the BBB and reach brain cells [7]. Different polyphenolic compounds display variable ability to accumulate in the CNS (central nervous system) depending on their properties related to their chemical structure [8,9]. Population aging and prolonged life span observed in developed countries contribute to an increase in the incidence ratio of chronic age-related neurodegenerative disorders, such as PD (Parkinson’s disease), AD (Alzheimer’s disease), or numerous forms of age-related dementia [10,11]. Dietary supplementation with neuroprotective plant-derived polyphenols might be considered an important element of healthy aging [12]. In adult humans, consumption of cocoa polyphenols improved cognition, mood, and visual functions, as well as sustained mental demand [13,14]. Studies on the role of long-time polyphenol consumption by adults reported improved language and verbal memory functions 13 years later [15]. Furthermore, in elderly people, the intake of plant polyphenols improves neurocognitive functions, slows the development of neurodegenerative diseases, or delays their onset [16]. In fact, a berry polyphenol-enriched diet is considered a strategy for memory improvement, especially during aging. In studies conducted on human beings, polyphenolic extracts of grapes and blueberry fruit were shown to improve memory functions in healthy elderly volunteers with lower levels of memory performance [17]. In experiments conducted on old adults consuming cranberry juice for over 6 weeks, the intervention did not result in successive cognition improvements. In this trial, however, study participants were not affected by cognitive decline [18]. Another study investigating the impact of berry and grape polyphenols, conducted on healthy people and participants with mild cognitive deficits, revealed significant improvement in cognition after 12 weeks of polyphenol intake [19,20]. Many polyphenols seem to be promising candidates as CNS therapeutics, based on the results obtained in in vitro studies [21,22]. However, these observations are often inconsistent with results from in vivo studies and clinical trials [23,24]. These discrepancies are due to, among others, inefficient transport across the BBB as well as decreased bioavailability and stability in the CNS [25]. Considering the issue of polyphenols and their metabolites’ permeability through the BBB, the existence of various specialized transporters, present on the luminal and abluminal sides of the barrier, should be taken into account [26].

Therefore, in this review, the latest achievements in studies (animal models and clinical trials) on the effect of polyphenols on brain activity and function are described. Moreover, the mechanisms of indirect and direct action of polyphenols on the CNS, with particular emphasis on the role of the BBB in these processes, are elucidated. Finally, different approaches to the improvement of the polyphenols’ delivery to brain tissue as an important condition to enhance the beneficial effect of these phytochemicals on the CNS in humans are presented.

## 2. Effect of Dietary Polyphenols on Brain Activity

The beneficial impact of plant polyphenols on the brain may be summarized by their role in increasing brain plasticity and related cognition improvement. Numerous studies investigate the impact of plant-derived polyphenols on brain cell metabolism, neutralization of reactive oxygen species, and cognitive functions related to signal transduction and neuronal plasticity [27,28,29,30]. As reversible MAO (monoamin oxidase) inhibitors, polyphenols are mood modulators and improve neuronal self-being by increasing dopamine, serotonin, and noradrenaline amounts in the brain tissue [31].

### 2.1. Impact of Various Polyphenols on Brain Functions—Animal Models

Studies on mice have shown that curcumin and piperine consumption decreases MAO-A and MAO-B-dependent monoaminergic neurotransmitters decomposition, which results in elevated serotonin and dopamine level in animal brain tissue, exhibiting natural antidepressant activity, as well as enhancing the effect of subthreshold doses of antidepressant drugs [31]. In another study curcumin in rat diet improved their reaction to stress restoring the hippocampal BDNF (brain-derived neurotrophic factor) and CREB (cAMP response element-binding protein) signaling [32]. What is more curcumin ingestion exhibited neuroprotective activity in rats after fluid percussion injury, where curcumin-rich diet caused normalization in BDNF level, and related CREB and synapsin1-dependent synaptic plasticity. In the brain polyphenols improve hippocampal neurogenesis, learning skills and memory. It was observed that polyphenol-rich diet boosts BDNF amount, thereby regulating the maintenance and formation of new synapses and improving memory formation [27,28]. As enhancers of BDNF level dietary polyphenols also contribute to the increased neurons survival during inflammation. Considering the CREB/BDNF-dependent neuronal activity of dietary polyphenols, promising results were obtained from experiments conducted on animals consuming berry juice [33]. In this study, blueberry polyphenols’ consumption was associated with improvement in spatial working memory and cognitive functions. Observed effects were related with enhanced hippocampal plasticity and CREB-dependent BDNF production [33]. Blueberry polyphenols consumption also improved object recognition in rats, which was linked with lower oxidative stress due to the lower NFĸB (nuclear factor kappa B) level in different brain areas [29]. Apart from berry juice polyphenols, also tea flavanols were able to improve brain functions in mice with cognitive deficits [34,35]. Animal studies have suggested the utility of plant-derived polyphenolic compounds in amyloid aggregation-related disorders like AD or PD. Experiments conducted on rats have shown dopaminergic neuroprotection provided by quercetin in rats. Its biological effect was to maintain the tyrosine hydroxylase amount in cytoplasm which, in turn, was supporting dopamine synthesis. Quercetin-induced reduction in ROS formation as well as decrease in proinflammatory cytokines’ synthesis and apoptosis ratio in brain tissue were also noticed [30]. This raises the possibility of using some polyphenols for slowing Parkinson’s progression. As reported by Hamaguchi et al. plant polyphenols may interfere with Aβ (amyloid beta) aggregation, mitigating AD pathology [36]. Resveratrol, a polyphenolic antioxidant enriched in grape, can cross the blood-brain barrier and exert protective effects against cerebral ischemic injury [37]. In developing mice received sevoflurane exposure (the most commonly used inhaled anesthetic in pediatric anesthesia), resveratrol pretreatment ameliorated cognitive impairment by modulating SIRT1-NF-κB pathway in microglia [38]. Animal studies investigating the action of plant-derived polyphenols on brain functions were summarized in Table 1.

### 2.2. The Impact of Plant-Derived Polyphenols on the Brain and Cognitive Functions

The randomized control trials focus on the impact of plant polyphenols on the human brain and check their role in improving the functions of a healthy brain, preserving memory and cognitive functions lost during aging, or mitigating the symptoms of neurodegenerative diseases (Table 2). Dietary polyphenols were reported to improve brain activity in depression. Curcumin from *Curcuma longa*, as well as eugenol, piperine, quercetin, and resveratrol, were proven to act as reversible inhibitors of MAO—an enzyme involved in serotonin degradation [44]. Thus, their use may improve the activity of serotonin-, dopamine-, or norepinephrine-utilizing synapses, contributing to mental well-being. Polyphenol compounds in the diet may modulate the activity of the human brain, contributing to reduced outcomes in neuropsychiatric disorders. In a randomized clinical trial study, a diet supplemented with bark-extracted polyphenols from French maritime pine significantly decreased ADHD (attention deficit hyperactivity disorder) symptoms in children, improving concentration and attention after a 4-week-long supplementation with Pycnogenol^®^ (Geneva, Switzerland) [45]. A plant polyphenol-rich diet not only mitigates the decline of cognitive functions during aging, but also these compounds are also considered helpful in the prevention and treatment of neuroinflammatory and neurodegenerative disorders. Numerous dietary polyphenols exhibit anti-inflammatory and antioxidant activity, which makes them potentially active in mitigating AD development [40,46,47,48,49,50,51]. However, the results of AD-related experiments are not clear. Numerous reports describe the impact of various polyphenols on APP (amyloid precursor protein) processing, aggregation, and toxicity of Aβ aggregates. Curcumin, myricetin, or gallic acid were reported to interfere with Aβ aggregation by inhibiting the nucleation and/or the elongation phase of Aβ aggregates formation [52,53,54,55]. Furthermore, numerous in vitro studies report numerous polyphenol activities with potent beneficial actions in AD. Curcumin and resveratrol were reported to reduce ROS generation, hyperphosphorylation of tau protein and improve SH-SY5Y cell viability in the presence of Aβ oligomers [21]. Thus, these polyphenols may be considered interesting agents supporting AD treatment, however, recent findings describe their pro-oxidative cytotoxic properties [24,55]. Nevertheless, dietary polyphenol intake seems to be an important factor in decreasing the risk of AD and dementia development during aging. An observational study conducted on the French population in the Bordeaux region indicates the importance of specific plant polyphenol-rich products’ consumption in significantly lowering the chance for dementia and AD development [56].

Intensive studies, in which plant-derived polyphenols were used as therapeutic agents useful in the treatment and prevention of PD, were carried out. In fact, it has been reported that dietary polyphenols present in Mediterranean and Asian diets decrease the risk of PD development [57,58]. Polyphenolic acids, especially gallic acid and its derivatives, were reported to significantly improve cells’ viability and reduce apoptosis, which resulted from 6-hydroxydopamine-induced oxidative stress and may be beneficial for PD [59]. It was reported that men consuming huge amounts of flavonoids and anthocyanins exhibit a significantly lower risk of PD development [57,60]. Beneficial activity in PD was also reported for quercetin [61]. Studies on the effectiveness of green tea polyphenols administration in mild PD gave confusing results. After 6 months of green tea polyphenol administration, the PD symptoms measured with a unified PD rating scale improved compared to the control, but surprisingly, no effect was observed after 12 months of green tea administration [23].

**Table 2:** Studies conducted on humans investigating the impact of plant-derived polyphenols on the brain and cognitive functions (interventional studies).

	Study Objective	Study Group	Study Description	Main Results	References
Interventional studies	To investigate the acute and subjective effects of cocoa flavanol (CF) consumption during mental demand	Healthy adults during mental demand	Randomized, controlled, double-blinded, balanced, three-period crossover trial; groups consuming 520 mg, 994 mg cocoa flavonol drinks, or matched control (0 mg)	Cognitive function improvement, attenuated mental fatigue	[14]
	To investigate the effect of acute cocoa flavonol consumption in dark chocolate on visual and cognitive functions	Healthy young adults	Randomized, single-blinded, order counterbalanced, crossover study; participants intake 720 mg of cocoa flavonols in dark chocolate or a matched amount of white chocolate, one-week interval between sessions	CF improved visual contrast sensitivity and reduced the time required to detect motion direction, but had no statistically reliable effect on the minimum proportion of coherent motion that could be detected; in terms of cognitive performance, CF improved spatial memory and performance on some aspects of the choice reaction time task	[13]
	Evaluation of the long-term association between polyphenol intake and cognitive performance	35–60-year-old women 45–60-year-old men	Participants in the SU.VI.MAX study (randomized, double-blind, placebo-controlled 8-year trial) were invited to perform 24-h dietary records every 2 months, records were randomly distributed across 2 weekend days and 4 weekdays in the year with all seasons and days covered, data collected with computerized questionnaires	Association between language and verbal memory improvement and high total polyphenol intake; catechins, theaflavins, flavonols, and hydroxybenzoic acids intake corresponded with better language and verbal memory performance, especially with episodic memory; negative association between executive functioning and dihydrochalcones, catechins, proanthocyanidins, and flavonols was observed	[15]
	Impact of chocolate, wine and tea flavonoids intake on cognitive performance	70–74-year-old participants, 55% women	Cross-sectional study	Study participants consuming chocolate, tea, or wine had significantly better mean scores in cognitive tests, lower prevalence of weak test scores; dose-dependent effects with a maximum of 10 g/day (chocolate) 100 mL/day (wine), linear for tea	[16]
Interventional studies	Evaluation of the effect of polyphenol extracts from grapes and blueberries on memory functions	60–70-year-old healthy participants	Bicentric, randomized, double-blind, placebo-controlled; study group received 258 mg of flavonols/day or placebo for 6 months	Improvement in verbal episodic and recognition memory tests; improved test results in groups with advanced cognitive decline in response to polyphenols intake	[17]
	Analysis of the short-term efficacy of cranberry juice intake on neuropsychologic functions in the elderly without cognitive deficits	50 participants, >60 years old, without cognitive deficits or dementia	Randomized, double-blind, placebo-controlled, conducted in parallel groups, 6-week study; participants randomly allocated to study groups obtained 245 g of cranberry juice (n = 25) or placebo (n = 25) daily; neuropsychologic tests before the interventions and after 6 weeks of the study	No significant actions were reported	[18]
	Impact of concord grape juice on neurocognition.	12 older adults with memory decline, dementia excluded	Randomized, placebo-controlled, double-blind trial, 12 weeks concord juice supplementation	Significantly improved verbal learning Increased verbal and spatial recall No effect on depressive symptoms or weight	[20]
	Impact of daily consumption of wild blueberry juice on neurocognition.	9 adults with early memory changes	Randomized, placebo-controlled, double-blind trial, 12 weeks of blueberry juice supplementation	Improved paired associate learning, word list recall, Trends suggesting reduced depressive symptoms (not significant)	[19]
	Effect of Pycnogenol on ADHD symptoms	61 children	Randomized, placebo-controlled, double-blind trial, duration 4 weeks, daily supplementation of pycnogenol in dose of 1 mg/kg; children examined before supplementation, 1 month after start, 1 month after end of the trial	1 month after starting, significant reduction of hyperreactivity in ADHD-affected children; improved attention, motoric coordination, and concentration 1 month after the end of supplementation: relapse of the symptoms	[45]
	Assessing safety, tolerability, and efficacy of green tea polyphenols	410 untreated participants with Parkinson’s disease, disease duration no longer than 5 years	Multicenter, double-blind, randomized, placebo-controlled, 12 month study, tests at the beginning, after 6 months and at the end of the study; not heavy tea drinkers were randomly allocated to groups obtaining 400, 800, or 1200 mg of green tea polyphenols daily, given orally in two doses	Contradictory results; beneficial effect observed after 6 months of the intervention was abolished after 12 months	[23]
Observational studies	Role of polyphenols intake profile in dementia development	1329 participants, mean age 78	Observational, nonintervention, 12-year follow-up study	Lower dementia and Alzheimer’s disease development risk was associated with a diet containing polyphenols from nuts, citrus, berries, leafy vegetables, soy, cereals, and olive oil accompanied by tea and red wine	[56]
	Examination of the role of flavonoids (and their subclasses) intake on the risk of PD development	49,281 men 80,336 women participants from the NHS (Nurses’ Health Study) and HPFS (Health Professionals Follow-up Study) programs	Progressive, nonintervention 20–22-year follow-up study Questionnaires among the studied groups	In the men’s group high flavonoids intake was linked with a 40% lower PD risk as compared with participants consuming low flavonoids quantities. Anthocyanins from berry consumption lower the risk of PD development	[60]

### 2.3. The Impact on Cognitive Functions in Humans

Many studies have revealed that a nutritional model based on increased consumption of antioxidants has the potential to ameliorate the cognitive impairment associated with neurodegenerative disorders (Table 3). It has been shown that diets rich in anti-inflammatory and antioxidant compounds, in combination with limiting the consumption of calories, reduce the decline in cognitive functions, the risk of cardiovascular disorders, and neurodegenerative diseases [17,62,63,64,65,66]. Moreover, in recent years, there has been a growing interest in the influence of a particular diet as a potentially modifiable factor in CNS disorders. It is now well known that the effect of certain nutritional patterns is a significant influencer in the protection against CNS impairment and nutrition in terms of brain protection, and this is receiving more and more attention [64,65]. After analyzing the prohealth effects of various eating patterns, it was postulated that their beneficial effects result from synergistic interactions between individual dietary components [66].

Randomized clinical trials have shown that the improvement of cognitive functions resulting from the consumption of foods rich in flavonoids is independent of age and health conditions (Table 3). Barfoot et al. determined the acute effect of a blueberry smoothie on particular aspects of executive functions in healthy schoolchildren (n = 54, aged 7–10), which is a critical period in the development of the CNS. It was noted that 2 h after the intervention, the reaction time of the modified attention network task (MANT) test in the study group was significantly shorter (compared to placebo) without affecting the accuracy of the response, and the verbal memory assessed by the auditory verbal learning test (AVLT) after consuming the blueberry cocktail was augmented. However, there was no difference between the groups in reading performance [60]. In turn, Whyte et al. assessed the acute effect of a smoothie of berries (blueberries, raspberries, and strawberries) on the improvement of executive functions and moods in young adults. The study enrolled 40 healthy subjects (aged 20–30) who consumed either a cocktail or a placebo. Cognitive functions were evaluated within 6 h of consumption. It was shown that cognitive performance during the working day (after 6 h) in the placebo group decreased due to fatigue, while in the study group, it was still maintained. Moreover, in the group receiving the cocktail, attention was significantly improved, both 2 and 4 h after consumption, compared to placebo. On the other hand, the blueberry intervention had no effect on the mood [63]. Whereas beneficial effects on cognition in the elderly were noted by Bensalem et al. and Godos et al. [64,67]. Bensalem et al. assessed the influence of polyphenol-rich grape and blueberry extract (PEGB) on memory. A total of 215 healthy elderly people (aged 60–70 years) who received 600 mg/day of PEGB or placebo were enrolled in the study; the duration of the intervention was 6 months. It was found that after 6 months of supplementation, cognitive functions decreased compared to the initial result, without any significant difference in the study and the control group. However, it was observed that after 6 months of PEGB consumption in the study group, recall memory was improved (*p* = 0.006) in comparison with placebo (without any differences in the baseline results in both groups), whereas, it was found that PEGB did not affect recognition or working memory. Importantly, the cohort was stratified into quartiles based on the initial primary outcome of the CANTAB (the Cambridge neuropsychological test automated battery), paired associate learning (PAL), and visuospatial learning and episodic memory tests; thus, a subgroup with cognitive impairment was distinguished. Within this subgroup, PEGB significantly improved cognitive performance compared to placebo. Thus, it was postulated that PEGB supplementation containing 58 mg of flavonoids; flavonols, flavan-3-ols, and anthocyanins (in particular quercetin, malvidin, chlorogenic acid, ferulic acid, gallic acid, and resveratrol) significantly enhance cognitive performance in elderly people with significant cognitive impairment [17]. The MEAL study, comprised of 2044 adult men and women from southern Italy, has proven that a higher dietary intake of flavonoids is positively linked with cognitive functioning. It has been demonstrated that a diet rich in flavonoids negatively correlates with cognitive dysfunction among the adult population (OR 0.39; 95% CI 0.15–1.00). Moreover, there is a positive correlation between the intake of flavonoid subclasses: anthocyanins, flavan-3-ols, flavonols, catechins, and cognitive functions. In the case of individual polyphenol compounds, an inverse association between the level of dietary intake and impaired cognitive status was found only for quercetin [64].

## 3. Direct and Indirect Action of Plant Polyphenols

Polyphenols improve brain functions by having a direct impact on cells and processes in the CNS. For a direct effect, polyphenolic compounds must be able to overcome the BBB and accumulate in the brain tissue. The exact mechanisms describing how the polyphenolic compounds cross the BBB are less known, although their presence in CSF (cerebrospinal fluid) has already been confirmed in humans [68]. In the CNS, polyphenols activate elements of intracellular signaling or modulate protein biology. The well-documented activity of polyphenolic compounds is the activation of CREB-dependent signaling, which in turn activates BDNF release in brain areas. An elevated level of BDNF improves memory and learning skills and protects from depression [27,69,70]. Polyphenolic compounds, through the induction of BDNF, have also been shown to strengthen adult hippocampal neurogenesis [67,71]. Plant polyphenols may also modify the metabolism of neuronal cells. Recently, Baron et al. described the direct impact of polyphenols from rosemary extracts on human neuroblastoma cells, reporting elevated glucose uptake in examined cell line cultures [72]. The observed polyphenolic activity was related to the Akt-independent activation of AMP (adenosine monophosphate)-protein kinase. Furthermore, polyphenol- or insulin-induced glucose uptake was statistically similar [72]. The described ability of polyphenolic compounds to increase insulin-independent glucose uptake by neurons is important for improving brain activity in age-related dementias, particularly AD, and additional studies addressing the transmission of bioactive polyphenols from examined extracts through the BBB and their effectivity should be carried out in animal models.

**Figure 1:**
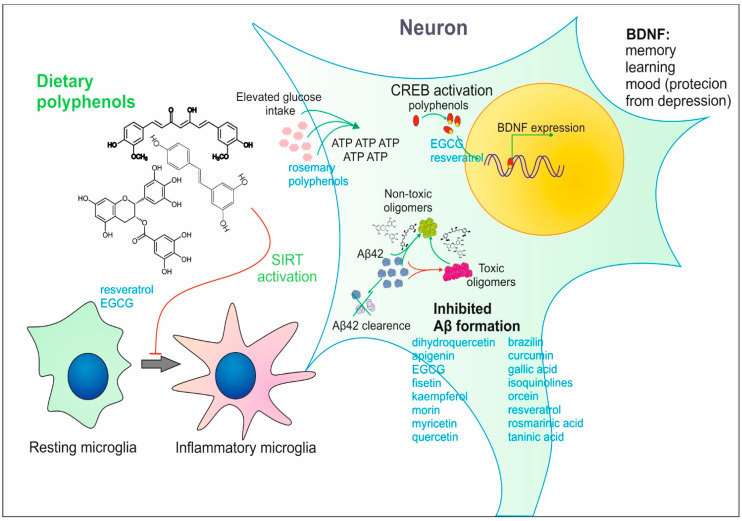
Direct action of plant-derived polyphenols on functions of the central nervous system.

Upgrowing data describe the SIRT1 (Sirtuin 1)-mediated mechanism of polyphenolic compound activity in the brain. Sirtuin 1 is an intracellular signal protein called a “metabolic sensor” and, through its deacetylase activity, regulates numerous molecular targets: the NFκB-dependent inflammation, p53 signaling-dependent apoptosis, histone acetylation/deacetylation, circadian rhythm, and FoxO (forkhead box O)-dependent resistance to oxidative stress. SIRT1 expression has been reported for cells in multiple tissues and organs [73]. Polyphenols or their derivatives are capable of allosteric activation of SIRT1 proteins and also upregulate the intracellular number of SIRT1 molecules. The exact mechanism describing how plant polyphenols increase SIRT1 levels remains unknown; however, it is hypothesized that SIRT1 expression is sensitive to reactive oxygen species, whereas polyphenols in cells act as antioxidants. In fact, NFκB suppression-dependent inhibition of the inflammatory response in the CNS caused by dietary polyphenols has been reported in numerous studies [74]. Recent findings describe the ability of resveratrol pretreatment to mitigate sevoflurane-induced cognitive impairment. Resveratrol administration reversed sevoflurane-inhibited SIRT1 activity, and also the M1 microglia activation skewed the M1/M2 balance toward M2 microglia. This finding opens the possibility of using resveratrol or other polyphenols to ameliorate the negative effects of commonly used anesthetics in pediatric anesthesia [38]. The ability to suppress M1 microglia activation and inflammation was also reported for the green tea catechin EGCG (epigallocatechin-3-gallate). In studies conducted on the BV2 cell line, EGCG was able to ameliorate the LPS (lipopolysaccharide)-induced microglia activation by decreasing the TLR4/NFkB (toll-like receptor 4/nuclear factor kappa B)-dependent inflammasome activation [22]. Ways of assessing the direct impact of dietary polyphenols on the CNS are summarized in Figure 1.

Apart from their direct action on cellular signaling, polyphenols inhibit protein aggregation in the brain tissue, protecting or delaying the outcome of aging-related disorders caused by protein aggregation. Baicalein, a flavonic polyphenol from *Scutellaria baicalensis* Georgi, exhibits neuroprotective activity in the suppression of Tau protein aggregate formation. The polyphenol molecules induced the dissolution of already formed Tau fibrils and caused the dissociation of captured oligomers [75]. Furthermore, oligomers were nontoxic and protected from Tau protein aggregation. Similar activities have also been reported for resveratrol [76], curcumin [55], and quercetin [77]. Plant flavonols also protect against Aβ aggregation in AD [69,70] or slow SOD1 (superoxide dismutase 1) aggregation in ALS (amyotrophic lateral sclerosis) [78].

There is also strong evidence describing the indirect impact of plant polyphenols on brain health. The intake of dietary polyphenols induces numerous improvements in vascular functions. Polyphenolic compounds are capable of lowering blood pressure and improving cerebral blood flow [79,80,81,82,83]. Intake of blueberry flavonoid polyphenols is linked with a decrease in NADPH (nicotinamide adenine dinucleotide phosphate)-dependent oxidase activity and an elevated level of circulating NO (nitric oxide) species [84]. A decrease in NO bioavailability is considered a cause of endothelial dysfunction, which in turn leads to atherosclerosis and stroke [85]. Thus, flavonoid polyphenols in the diet exhibit neuroprotective activity through the improvement of cerebral blood flow.

Polyphenols act on the brain endothelial cells and improve the BBB’s integrity and reduce inflammation, thus protecting the brain from additional injury during stroke or autoimmune diseases. Green tea polyphenols are able to reduce ischemia-elevated BBB permeability, as shown by Zhang and others in the rat occlusion model of cerebral ischemia [39]. Recent studies with commercially available extracts of plant-derived polyphenols, including Neumentix^®^, confirmed their beneficial action on BBB integrity and ability to reduce the inflammatory response in mouse brain tissue after middle artery occlusion [40]. Polyphenol-induced sealing of the BBB during ischemia can mitigate the development of brain inflammation. Furthermore, increased BBB permeability contributes to AD pathogenesis since the Aβ deposits lead to barrier disruption. Recent findings by Xiong and others describe the ability of polyphenols from lychee seeds to protect the BBB from Aβ (25–35)-induced disruption, which could be helpful for improving AD treatment [41].

**Figure 2:**
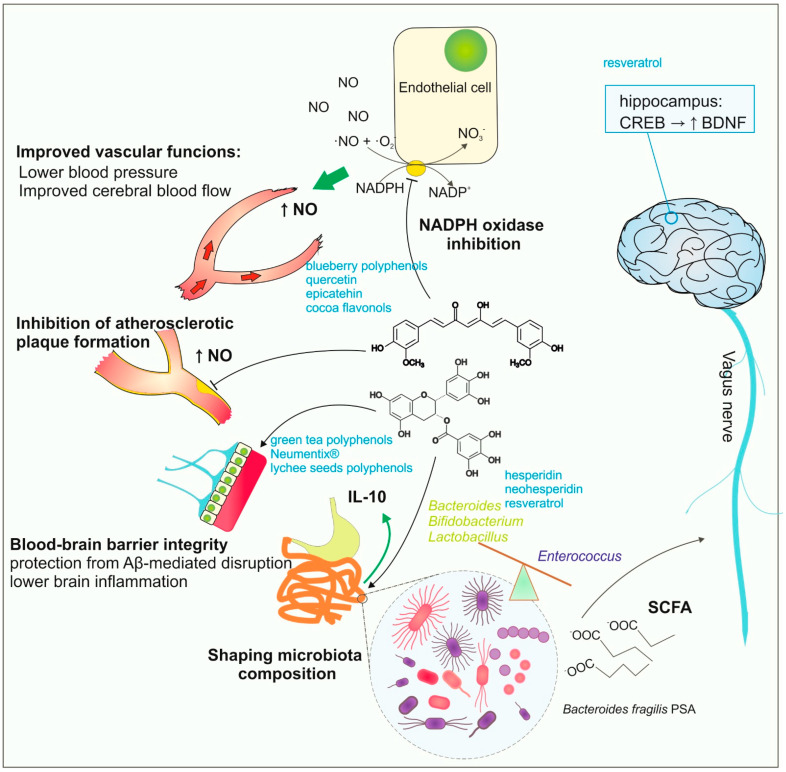
Indirect effects of plant-derived polyphenols on the functions of central nervous system.

Actually, the widely discussed activity of polyphenols is their impact on the composition and metabolic activity of human microbiota, and the axis of polyphenols-gut microbiota-brain interactions is extensively discussed [86]. The gut microbiota regulates the activity of the immune system by providing metabolites such as SCFA (short-chain fatty acids) or immunomodulatory polysaccharides (i.e., polysaccharide A from *Bacteroides fragilis*). Capsular polysaccharide A (PSA) is an inducer of IL-10 production, and microbiota with an elevated *B. fragilis* ratio is effective in preventing inflammation and autoinflammatory disorders [87,88,89]. Two-dimensional interactions between the central nervous system and gut microflora occur via the stimulation of the vagus nerve [90,91]. Dietary polyphenols are able to modulate the composition of gut microbiota, and bacterial microflora may digest or modify polyphenolic compounds, which determines the content of polyphenols available for organisms [92,93]. Polyphenols from citrus fruits, such as hesperidin or neohesperidin, were shown to restore the diversity in gut microbiota, increasing the number of *Bacteroides*, *Firmicutes*, or *Lactobacillus* species [94,95,96]. The ability to modify neural activity via the intestinal microbiota was also reported for resveratrol. In animal studies, a resveratrol-enriched diet increased microbiota biodiversity and the number of *Lactobacillus* and *Bifidobacterium* species, whereas the genus Enterococcus decreased [97]. Gut bacteria metabolize resveratrol mainly to dihydroresveratrol, 3,4′-dihydroxybibenzyl, and 3,4′-dihydroxy-*trans*-stilbene, and also increase resveratrol’s bioavailability [98,99,100]. Resveratrol also stimulates the 5-hydroxytryptamine synthesis in the gut, and it was proven to induce BDNF synthesis in the hippocampus via 5-HT (5-hydroxytryptamine)-dependent induction of CREB signaling [42]. Actually, it is clear that the functional state of intestinal microbiota and its composition should be taken into consideration as an important agent modifying the potential properties of polyphenolic compounds by their metabolism and changing their bioavailability. On the other hand, numerous dietary polyphenols act as prebiotics, shaping the composition and bioactivity of the human microbiota and contributing indirectly to the commensal bacteria-driven impact on nervous system activity. The indirect impact of plant-derived polyphenols on the brain is summarized in Figure 2.

## 4. Availability of Polyphenols in the Brain Tissue

The blood-brain barrier is a structure formed by brain endothelial cells, astrocyte processes, and pericytes. The presence of the BBB limits and controls the trafficking of chemical substances and immune cells from the blood to the brain parenchyma. Selectivity of brain endothelial cell-mediated transport from the blood determines brain homeostasis. The presence of BBB reduces the bioavailability of dietary polyphenols and their metabolites for brain tissue. Little is known about the transport of dietary polyphenols and its limitations through the BBB; however, it is suggested that passive and active mechanisms may underline the polyphenols’ movement across the BBB.

### 4.1. BBB Reduces the Direct Impact of Dietary Polyphenols on Neurons

Recent years’ findings suggest the selectivity of endothelial transport for polyphenol derivatives depends on their structure and chemical modifications. Figueira and co-workers described the differences in the transport of berry juice polyphenols’ derivatives across the BBB depending on their structure and chemical modifications. They observed enhanced transport of methylated or sulfated gallic acid and catechol derivatives across the BBB, whereas the transport of similarly modified pyrogallol derivatives was less effective. The authors, in their elegant work, have also described the activity of BCRP (breast cancer resistance protein)-transporter proteins belonging to ABC (ATP-binding cassette)-type efflux transporters in human immortalized brain endothelium cells, actively limiting the accumulation of polyphenol derivatives in the brain tissue [8]. Similar efflux activity of the BCRP transporter was also described to limit quercetin accumulation [101]. Finally, brain endothelial cells were also noticed to metabolize polyphenols during their transport from plasma, which may contribute to their lower availability for neurons [8]. More lipophilic polyphenols or their metabolites are supposed to be more motile through the endothelial cells depending on their passive permeation; however, the presence of specific efflux activity should always be considered.

### 4.2. Polyphenol Transport across the Blood-Brain Barrier

The major obstacle to the application of polyphenols in the prevention of neurodegenerative disorders is their bioavailability. The presence of the BBB limits the transport of many compounds into the brain; thus, it is the key regulator of the entry and retention of polyphenols in the brain. The BBB is a highly selective barrier protecting the CNS from toxic compounds and pathogens, isolating the periphery from the brain [102]. Transport across the BBB occurs via six main routes: paracellular diffusion, lipid-mediated diffusion, carrier-mediated transport, receptor-mediated transcytosis, absorptive-mediated transcytosis, and active efflux transport [103]. The permeability of BBB endothelial cells to polyphenols is mainly influenced by the expression of efflux transporters and solute carriers [104].

The permeation of polyphenols across the BBB has been studied by Youdim and co-workers [101,105]. They suggested that polyphenols’ ability to cross the transmembrane in vitro depends on the degree of their lipophilicity, where less polar polyphenols or their metabolites (e.g., methylated molecules) have greater brain uptake than the more polar ones. However, it is not clear whether the main route of polyphenol transport into the CNS is simple diffusion or carrier-mediated transport [106]. It was also observed that polyphenols are able to modulate the activity of some ABC drug transporters, which are efflux pumps. Flavonoids have been shown to inhibit the ABC transporters [107,108,109]. Moreover, some flavonoids are themselves substrates for various ABC transporters, such as P-gp (P-glycoprotein), MRP2 (multidrug-resistant protein type 2), and BCRP [110]. Such interactions dramatically limit their bioavailability. Youdim and coworkers have shown that quercetin can cross the BBB when coadministered with a P-gp or BCRP inhibitor. It was suggested that this compound was able to enter the CNS, possibly by passive diffusion due to its hydrophobicity, but was then exported by efflux transporters [101]. Although most polyphenols are ligands for efflux transporters, they may still penetrate the brain in substantial amounts to exert protective function, as was shown for resveratrol [111].

Numerous in vitro and in vivo studies have pointed out that polyphenols’ ability to cross the BBB is affected by their chemical modifications, e.g., methylation, sulfation, or glucuronidation [9,112]. Methylation or glucuronidation of selected polyphenols or their metabolites increases their permeation across the BBB models in vitro [9]. Studies utilizing animal models have revealed that polyphenols accumulate in the brain in a nonregion-specific manner. Moreover, it was shown that these compounds are able to cross the BBB and accumulate in brain tissue independently of their route of administration [113,114,115,116]. Among others, curcumin has been shown to be transported to the CNS, where it induces neuroprotection by antiapoptotic mechanisms and mitochondrial protection [51,117,118]. Additionally, resveratrol was shown to cross the BBB and incorporate itself into the brain tissue [37]. Moreover, phenolic acids, such as caffeic acid, accumulate in the CNS and exert neuroprotective effects during cerebral ischemic injury [43,119]. The ability of caffeic acid to cross the BBB has also been observed in the CSF of patients with neurological disorders [68].

### 4.3. Evidence from Human Studies on the Passage of Polyphenols through the BBB

The use of dietary patterns with foods rich in flavonoids may prevent cognitive impairment due to the well-known multimolecular mechanisms of their action. What is more, based on recent discoveries, an impact on neuronal cells due to passing the BBB by flavonoid metabolites is considered a possible mechanism through which certain flavonoids could exert a direct neuroprotective effect [101]. However, epidemiological evidence is still limited, and there are discrepancies between in vitro and in vivo evidence on the neuroprotective role of flavonoid compounds. In vitro models are an important tool for assessing the penetration ability of xenobiotics across the BBB, but they do not fully reflect the physiological conditions. The main limitation is the inability to determine the contribution of efflux transporters involved in restricting the passage of compounds into the brain from the blood. Thus, the use of only in vitro models is insufficient to accurately determine both qualitative and quantitative penetration of substances into the human brain [25]. Due to highly invasive methods of obtaining samples from the CNS, there is a large deficit in research concerning the concentration of polyphenols in the cerebrospinal fluid (CSF) and brain of humans. Thus, most of the studies on the CSF bioavailability of phenolic compounds are based on in vitro and animal model studies, but only human data can fully provide knowledge on the usefulness of using these compounds in CNS diseases [25].

The current state of knowledge suggests that the passage of polyphenols and their metabolites through the BBB depends on their lipophilicity [101]. However, to establish the necessary conditions for human BBB penetration, it is still crucial to define their active forms, the activity locations, and the mechanism of neuroprotective actions (Table 4) [105].

High therapeutic hopes are associated with the use of resveratrol in the prevention of neurodegenerative diseases. Particularly important neuroprotective properties of resveratrol are related to anti-inflammatory, -oxidative, -apoptotic, and -aging activities [124], which have been investigated in several clinical trials [125,126,127,128,129]. It has been demonstrated that the administration of resveratrol was associated with a dose-dependent increase in cerebral blood flow and deoxyhemoglobin levels in healthy adults [125], improvement of memory performance through augmentation of glucose metabolism, and increased functional connectivity of the hippocampus in the overweight elderly [126], as well as enhancement of cognition function [127]. Moreover, resveratrol supplementation reduced the level of glycosylated hemoglobin A1c, retained the volume of the hippocampus, and improved resting-state functional connectivity in patients with mild cognitive impairment [129]. In preclinical studies, it has been observed that resveratrol penetrates the BBB [37]. This was confirmed in phase II multicenter randomized controlled trial that investigated the effect of 52-week supplementation with resveratrol (500 mg/day, with a dose increase of 500 mg every 13 weeks, final dose 2000 mg/day) in patients with mild-to-moderate AD (n = 119). Resveratrol, as well as its major metabolites: 3-O-glucuronidated-resveratrol (3GRES), 4-O-glucuronidated-resveratrol (4GRES), and 3-sulfated-resveratrol (SRES), were detected in the CSF and thus penetrated the BBB. After a 52-week-long intervention, the mean CSF concentrations of resveratrol, 3GRES, 4GRES, and SRES for the entire population were 0.45 ± 0.14 ng/mL, 8.3 ± 1.3 ng/mL, 11.2 ± 1.7 ng/mL, and 12.6 ± 1.6 ng/mL, respectively. Moreover, it has been found that the concentration of Aβ40 (the 40-amino acid isoform of β-amyloid being a diagnostic biomarker of AD), both in plasma and CSF, showed a smaller decrease in the study group as compared to the control. Whereas there were no differences between the groups in the level of Aβ42 (the 42-amino acid isoform of β-amyloid being a diagnostic biomarker of AD) in CSF, tau in CSF, phospho-tau 181 in CSF, as well as in functional tests [121]. Similarly, the recent study by Gu et al. confirmed the neuroprotective effect of resveratrol at a dose of 500 mg/day in patients with AD (mild-to-moderate), after 52 treatments. They demonstrated that this polyphenol regulated a progressive decline in Aβ40 levels in both CSF and plasma compared to placebo as well as based on an MRI scan; they showed that the brain volume was reduced in the test group compared to the placebo (*p* = 0.011). Moreover, it was observed that in the group receiving trans-resveratrol, the worsening in motor performance (based on the Alzheimer’s Disease Cooperative Study Activities of Daily Living Scale) was significantly reduced [130]. Although high-dose resveratrol is safe, well-tolerated, penetrates the BBB, and has direct effects on the CNS [121,130], the interpretation of changes in biomarker levels should be approached with caution. Due to small, pilot studies, it is necessary to conduct multicenter randomized trials that will allow for establishing the benefits of resveratrol in neurodegenerative disease.

The beneficial effects of curcumin on CNS diseases have been demonstrated in preclinical and clinical studies [47,122,131]. Its neuroprotective effect is mainly based on strong antioxidant and anti-inflammatory properties, as well as inhibiting the aggregation of misfolded proteins. In clinical studies, it has been observed that supplementation with curcumin, both singly and in combination therapy, mainly with coenzyme Q7 and omega-3 acids, reduces the severity, duration, and frequency of migraine attacks [132,133], the occurrence of motor disorders in Parkinson’s disease [129], and improves the clinical status of patients with amyotrophic lateral sclerosis [131]. The only available study assessing the levels of curcumin and its metabolites in CSF has not demonstrated a beneficial effect in AD patients. Ringman et al., in the randomized controlled trial—the Curcumin C3 Complex (^®^)—assessed the effect of oral curcumin supplementation in patients with mild-to-moderate AD (n = 36). The studies lasted 24 weeks, with an open-label extension of 48 weeks. Patients received curcumin at a dose of 2 g/day, 4 g/day, or placebo for 24 weeks, then for the next 24 weeks, patients in the curcumin group continued the dose, while patients in the placebo group received curcumin at a dose of 2 g/day or 4 g/day. The purpose of this study was to investigate both curcumin’s effectiveness and bioavailability. They found that curcumin was well tolerated, but no clinical or biochemical improvement was observed. In contrast, in a pharmacokinetic study using liquid chromatography/tandem mass spectrometry, they showed that both native curcumin, tetrahydrocurcumin, and their glucuronidated metabolites were absent in CSF, suggesting that it does not penetrate the BBB [114]. The discrepancy in data on the beneficial effects of curcumin on the CNS implies a further examination of its neurorestorative activity, with particular emphasis on the pharmacological profile in humans. On the other hand, in vitro and preclinical studies have shown that quercetin, the most abundant naturally occurring flavonoid, has a neuroprotective effect through its antioxidant, anti-inflammatory, and cell toxicity-inhibitory activity [115,132,133,134]. However, the beneficial effects of quercetin in CNS diseases have not been confirmed in clinical trials because the limitation may be the low cerebral bioavailability of quercetin [135,136]. Nevertheless, intensive research on increasing the bioavailability of quercetin prompted Ishisaka et al. to investigate the cerebral localization of the main dietary metabolite of quercetin—quercetin-3-O-glucuronide (Q3GA). In a *post-mortem* study using human brain tissue with or without infarction using the immunohistochemical method, significant immunoreactivity was found in the cells of the choroid plexus, which form the structural basis of the blood-CSF barrier, and in foamy macrophages in recent stroke brains. The results of the immunohistochemical study implied an in vitro examination of the consequences of Q3GA cellular accumulation using brain capillary endothelial RBEC1, macrophage-like RAW264, and microglial MG6 cells. It has been demonstrated that immune cells may be a potential target of Q3GA because greater accumulation of Q3GA was observed in MG6 and RAW264 cells than in RBEC1. Importantly, this study noted that in MG6 and RBEC1 cells, Q3GA was deconjugated to its native and methylated forms. In addition, it has been found that the deconjugated forms in LPS-treated macrophages, as opposed to Q3GA, had anti-inflammatory effects by inhibiting the JNK pathway (c-Jun N-terminal kinase). Thus, it has been observed that quercetin glucuronide penetrates the BBB in humans and may accumulate in some types of immune cells [137]. Grabska-Kobylecka et al. showed that some polyphenols can pass through the BBB in humans. They examined the presence of 12 phenolic compounds in CSF, and collected data for diagnostic purposes from 28 patients (age 46 ± 16 years; 18 women and 10 men) with neurological diseases (multiple sclerosis, polyneuropathy, mononeuropathy, meningitis, brain tumor, epilepsy, amyotrophic lateral sclerosis, and Friedreich’s ataxia). Additionally, 30 min prior to the lumbar puncture, plasma was collected from each patient, and both plasma and CSF were assembled after an overnight fast. The identification of polyphenols in CSF and plasma was performed by combining solid-phase extraction with high-performance liquid chromatography. It has been shown that caffeic acid, homovanilic acid, and 3-hydroxyphenylacetic acid were present in CSF and plasma, while hippuric acid, 3,4-dihydrobenzoic acid, and dihydrocaffeic acid were detected in plasma only [68]. The sources of caffeic acid, homovanilic acid, and 3-hydroxyphenylacetic acid can be some vegetables and fruits, and they can also be metabolites of more complex polyphenols ingested. Nevertheless, homovanilic acid and 3-hydroxyphenol acetic acid can also be produced endogenously in the brain as products of catecholamine metabolism [134,135,136,137]. However, according to the current state of knowledge, caffeic acid is not produced endogenously, and its presence is indicative of an exogenous origin. Importantly, in the above study, no correlation between the concentration of phenolic acids in plasma and CSF was found, which may also suggest that the penetration of polyphenols through the BBB is not a result of facilitated or passive transport [68]. In turn, Zini et al. did not find detectable amounts of flavan-3-ols and their metabolites in CSF 2 h after green tea intake, while in the plasma collected 1 h after consumption, flavan-3-ol methyl, glucuronide, and sulfate metabolites: (2)-epigallocatechin glucuronide, (2)-epicatechin glucuronide, (2)-epicatechin sulfate, and methyl-(2)-epicatechin sulfate were identified by HPLC-MS2 analysis. The study included six patients, aged 34–61 (three women and three men), who had a lumbar puncture performed for diagnostic purposes due to the suspicion of multiple sclerosis. This study did not confirm green tea flavan-3-ol’s direct effect on the CNS. However, as the authors conclude, a serious limitation of this study is the small size of the group and a single dose of green tea [120].

An important issue in the direct action of polyphenols on the CNS is not only their penetration through the BBB, but also their brain metabolism and localization. However, as already mentioned, for methodological reasons, conducting mechanistic research in humans is extremely difficult, and knowledge on this subject is very limited. Nevertheless, human studies are crucial to unequivocally determining the effectiveness of antioxidant use in the prevention and treatment of CNS diseases.

## 5. Nanotechnological Solutions to Improve the Bioavailability of Polyphenols in the Brain

For therapeutic use, sufficient quantities of polyphenols must cross the BBB and reach the brain tissue in active form. The bioavailability of polyphenols is low. The main factors that limit this process and have an effect on therapeutic efficacy are: selective permeability across the BBB (dependent on their stereochemistry, interactions with efflux transporters, the degree of lipophilicity, the form of administration, and synergistic effects), gastrointestinal transformations, poor absorption, rapid hepatic and colonic metabolism, and systemic elimination [137]. The most usual oral administration also conflicts with bioavailability. Thus, phenolic compounds have inadequate bioavailability for human applications to have any beneficial effects. In addition, since polyphenols are sensitive to a great number of physiological conditions, for effective clinical applications, their stability must be improved. In recent years, new strategies have been attempted in order to exert cognitive benefits and neuroprotective effects. Converting polyphenols into nanostructures is one of the theories proposed to enhance their bioavailability.

### 5.1. Nanoencapsulation

To improve the bioavailability of phenolic compounds, nanoencapsulation technology can be used. The encapsulation process is the packing of small active particles (solid, liquid, or gas) into a matrix or shell, which is a secondary material [138]. That way, particles with diameters ranging from 1 to 1000 nm are formed. In such small capsules, the surface-volume ratio is increased. Nanoencapsulation provides significant protection against drastic conditions and allows for control of the release rate at the absorption site. The solubility of the encapsulated end product is improved, as well as toxicity to peripheral organs is reduced, and the degradation process in the gastrointestinal tract can be minimized. Other features of nanoparticles that should be emphasized are: nonthrombogenic, nonimmunogenic, and noninflammatory. It is essential that the encapsulation process does not affect or alter the medication’s properties. The following nanoscale delivery systems can be used to encapsulate polyphenols: nanocapsules, nanospheres, micelles, cyclodextrins, solid lipid nanoparticles, and liposomes [139].

### 5.2. Natural Nano-Carriers

Phenolics can be nanoencapsulated by natural nanocarriers, such as cyclodextrins, chitosan, casein, or nanocrystals. The dietary bioflavonoid quercetin can reduce inflammatory processes and exert neuroprotective effects when administered in vivo. The main anti-Alzheimer’s disease properties of quercetin include the inhibition of Aβ (amyloid-β) aggregation [140] and tau phosphorylation [141]. Quercetin, characterized by low solubility and poor absorption, is extensively metabolized in the gut after oral administration in humans. It has low BBB penetrability and limited bioavailability [142].

In order to improve its permeation across the BBB and its bioavailability to enhance neuroprotective effects, Testa and coworkers formulated b-cyclodextrin-dodecylcarbonate-based nanoparticles containing quercetin [49]. Cyclodextrin is a cyclic oligosaccharide in which 6, 7, or 8 glucopyranose units are joined by a glycosidic bond. The bioavailability of such a product is improved, as well as stability and other functional properties [143]. Such nanoparticles were used in an in vitro study on human neuroblastoma SH-SY5Y cells. Quercetin-loaded nanoparticles were noncytotoxic, and their anti-inflammatory effect in SH-SY5Y cells was stronger than that of free quercetin. The new therapeutic approach applied by the authors may allow for more effective use of the neuroprotective properties of quercetin for preventing/reducing AD progression. The study conducted by the Aluani group determined the neuroprotective activity of quercetin encapsulated in chitosan-alginate nanoparticles in two in vitro models: H_2_O_2_ induced oxidative stress in human neuroblastoma cells SH-SY5Y and 6-hydroxydopamine (6-OHDA) induced neurotoxicity in rat brain synaptosomes [144]. Naturally occurring polysaccharide, chitosan, is known for its high biodegradability, biocompatibility, and low toxicity. Quercetin-loaded nanoparticles, especially chitosan-based ones, were protective against oxidative stress in both models of neuronal toxicity. Thus, limitations associated with the clinical use of quercetin could be overcome, and its neuroprotective effect could be enhanced due to its incorporation into chitosan-based nanoparticles.

The first study investigating the protective and therapeutic effects of quercetin nanoparticles (QNPs) in an animal AD model was performed by Rifaai et al. [145]. AD was induced in rats by the oral administration of AlCl_3_. AlCl_3_ application triggered degenerative effects in the rat’s hippocampus, which were minimized by QNPs used as prophylaxis along with AlCl_3_. In addition, the AlCl_3_-induced neurofibrillary tangles and amyloid plaque formation were almost abolished in the same group of animals, confirming the prophylactic effect of QNPs in the AD model. Additionally, proliferation in the hippocampal dentate gyrus was preserved when rats were treated with QNPs. The authors demonstrated that the nanoparticle form of quercetin increased its bioavailability to the affected neurons and reduced the damaging effect of AlCl_3_ on hippocampal neurons at the molecular, cellular, and subcellular levels.

To induce the bioavailability and solubility of quercetin, Jajin et al. used the magnetic targeting of superparamagnetic nanoparticles [146]. The authors developed quercetin-conjugated superparamagnetic iron oxide nanoparticles (QT-SPIONs) and tested their effects on AD-like symptoms induced by AlCl_3_ in a rat model. What they discovered was that QT-SPION conjugates in all experiments were significantly more effective than quercetins alone and led to better bioavailability for quercetins. The entrance of quercetin into the brain tissue in the conjugate form was increased. QT-SPION augmented the expression levels of antioxidant enzymes (SOD1, CAT, and GPX1) reduced iNOS, along with an increase in the expression level of the antiapoptotic gene (BCL2) and a decrease in the proapoptotic gene (BAX). Experiments on rats with the application of aluminum to induce memory impairment have shown that treatment with QT-SPION recovered the memory impairment developed by AlCl_3_ and the appearance of AD-like symptoms. A significant reduction in the expression level of the amyloid precursor protein gene in the group treated with QT-SPION was also demonstrated, which proves the potential of QT-SPION to inhibit AD development and progression caused by aluminum exposure. The obtained results make quercetin-conjugated nanoparticles, a nanosized delivery system, able to penetrate the BBB with beneficial effects on neurodegeneration, especially effective in preventing the progression of AD symptoms in long-term usage.

### 5.3. Solid Lipid Nanoparticles (SLNs)

The most promising tool to aid in therapy for the treatment of AD is solid lipid nanoparticles (SLNs), a colloidal drug carrier system composed of a hydrophobic lipid core composed of an emulsion (oil in water) with lipids. The solid core, coated with aqueous surfactants, contains the drug, which is dissolved or dispersed [137]. For brain applications, the size of SLNs should not exceed 200 nm to cross the tight endothelial cells of the BBB, thus ensuring a high degree of tissue penetration.

The most important mechanisms making SLNs an attractive delivery system include low intrinsic cytotoxicity, the ability to bypass liver and spleen filtration, increased penetration to the BBB, the controlled release of the incorporated drug, and high stability [147,148]. Loureiro et al., in experiments on human brain-like endothelial cells, worked on developing an efficient system to increase the bioavailability of resveratrol, a natural polyphenolic flavonoid with known neuroprotective effects [149]. They took advantage of well-characterized morphologic lipid nanoparticles functionalized with an OX26 monoclonal antibody with the ability to bind cells expressing the transferrin receptors, TfR, present in the BBB. Such nanostructures were used as carriers for resveratrol, grape skin, and grape seed extracts. The authors demonstrated that encapsulated extracts were effectively transported into the brain, where the formation of Aβ(1–42) aggregates was inhibited, potentially influencing the progression of Alzheimer’s disease. The authors proved that the functionalization of SLN by coupling with the OX-26 antibody allows for targeting polyphenols or other drugs in the brain, where such nanoparticles are stable for at least one month and are taken by BBB cells.

Solid lipid nanoparticles were also used by Sandhir and colleagues to encapsulate curcumin (C-SLNs), and their neuroprotective efficacy was evaluated as an intervention in Huntington’s disease [150]. A rat model of 3-nitropropionic acid-induced HD in C-SLN-treated animals showed a notable increase in the activity of mitochondrial complexes, cytochrome, and reduced GSH levels. Additionally, reductions in mitochondrial swelling, lipid peroxidation, protein carbonyls, and ROS were observed when compared to nontreated rats. A significant improvement in neuromotor coordination has also been noted. These results proved that C-SLNs developed by this group have the potential to ameliorate the mitochondrial impairments in HD and could provide a potential therapeutic intervention in other neurodegenerative disorders.

Kundu et al. demonstrated in a study conducted with the use of curcumin and piperine-coloaded glyceryl monooleate (GMO) lipid nanoparticles coated with various surfactants demonstrated that such dual drug-loaded NPs were able to cross the BBB in a Parkinson’s disease mouse model [151]. The authors observed in in vitro experiments that alpha S protein aggregates inhibition, reduces cytotoxicity, oxidative stress, and apoptosis, and activates autophagy-mediated protein degradation induced by dual drug-loaded GMO nanoparticles compared to their native counterparts. Moreover, rotenone-induced motor coordination impairment was restored, and dopaminergic neuronal degeneration was restrained in a PD mouse model. In this case, a nanodelivery system is a promising tool to deliver drugs to the brain and enhance the bioavailability of multiple therapeutic agents for effective PD management.

### 5.4. Polymeric Nanoparticles

Polymeric nanoparticles are composed of a solid core of a dense polymer, natural (proteins and polysaccharides) or synthetic (poly(lactide), poly(lactic acid) PLA, poly(glycolic acid) (PGA), poly(d,l-lactide-co-glycolic acid) (PLGA), poly(alkyl cyanoacrylate) (PACA) [152]. The selection of an appropriate polymer for the production of nanoparticles depends on the needs of a specific application and the characteristics of the drug being introduced [153]. The encapsulated lipophilic drug can be solubilized in an oil or aqueous core surrounded by the polymeric shell (nanocapsules), dispersed throughout the polymeric network, or located on the surface of the polymeric matrix (nanospheres) [154]. A promising strategy to reduce degradability and increase the effective delivery of phenolics could be nanoencapsulation with biopolymeric nanoparticles, e.g., poly (lactic-co-glycolic acid) (PLGA). PLGA, commonly used in nanomedicine, is a synthetic biocompatible copolymer composed of lactic and glycolic acid polyesters, approved by the Food and Drug Administration (FDA, Silver Spring, MD, USA) for therapeutic use in pharmaceutical products [155]. The possibility of forming nanoparticles, micelles, and microspheres, together with their complete biodegradability and well-tolerable properties, makes them a tool for controlled, targeted delivery of small drugs into the brain through different transport mechanisms [156].

There are several studies reporting the use of polyphenols encapsulated with PLGA nanopolymer as drugs transported to the brain for the treatment of brain diseases, especially Alzheimer’s and Parkinson’s disease. Among them, nanoparticles loaded with curcumin drew the attention of researchers. Mathew and coworkers synthesized water-soluble curcumin nanoparticles encapsulated in PLGA. The authors found by using an in vitro AD model that nanocurcumin particles bound to Aβ aggregates and mediated their dissociation, along with antioxidative properties and noncytotoxicity [157]. Another study by Mathew et al. showed that functionalized curcumin-loaded PLGA nanoparticles conjugated with Tet-1 peptide, known for its high affinity for neurons, were also able to disaggregate amyloid proteins. The targeting of the nanoparticles with the Tet-1 peptide enhanced their uptake by neuronal cells. Curcumin-PLGA-Tet-1 nanoparticles retained their antioxidant properties and were not cytotoxic [158]. The research of this group proved that nanoformulations with encapsulated curcumin may have a beneficial impact in order to address problems related to the poor bioavailability and pharmacokinetics of this antioxidant. Another group took advantage of the curcumin-encapsulated PLGA nanoparticles. In a study conducted on neural stem cells (NSC) and adult rat brains, Tiwari and colleagues demonstrated that Cur-PLGA-NPs are able to induce NSC proliferation as well as neuronal differentiation as compared to bulk curcumin, which proves that PLGA nanoparticles internalize into the NSC and nanocurcumin reaches the brain [159]. The authors proved that curcumin-loaded nanoparticles induced neurogenesis, as confirmed by the experiments carried out with the use of an amyloid β-induced rat model of AD. The results also demonstrated that neurogenesis was mediated by activating the canonical Wnt/beta-catenin pathway. PLGA nanoencapsulation could be a promising approach to delivering curcumin to the brain.

Additionally, Ray and coworkers studied the properties of a polymeric nanoparticle formulation of curcumin, NanoCurc, in in vitro and in vivo experiments and demonstrated its neuroprotective effect [160]. In vitro, NanoCurc-treated human SK-N-SH cells were protected from H_2_O_2_-mediated ROS insults. In vivo, NanoCurc was injected intraperitoneally in athemic mice, and following such treatment, significant curcumin levels were observed in the brain of this cohort versus controls, together with decreased levels of H_2_O_2_, caspase activity, and increased glutathione concentrations. This is another study whose results confirm the increase in curcumin’s bioavailability when used in a water-soluble encapsulated form. The potential of NanoCurc to protect, preserve, and rescue human neuronal cells against oxidative damage may have implications in the treatment of neurodegenerative diseases. In turn, Gosh et al. investigated PLGA-nanoencapsulated quercetin as a drug carrier for oral treatment of ischemia-reperfusion-induced neuronal damage in young and old Swiss Albino rats [161]. Such therapy resulted in an improvement in neuronal count in the hippocampal subfields, where massive damage to neuronal cells was observed after three days of continuous reperfusion following ischemia. Moreover, the downregulation of iNOS and caspase 3 activities was demonstrated as a result of oral nanoencapsulated quercetin. The delivery of this potent herb-origin antioxidant to the brain may protect against oxidative stress that causes ischemic neuronal damage. Sun and coworkers tested PLGA-functionalized quercetin nanoparticles (PLGA@QT) and investigated their effect on the bioavailability of this potent flavonoid to be used in AD treatment [162]. First, the authors confirmed in an in vitro and in vivo cytotoxicity assay on the neuronal cell line SH-SY5Y and female BALB/c nude mice that such quercetin nanoformulations are nontoxic. Second, PLGA@QT inhibited and disassembled Aβ42 fibrils. They also determined that PLGA@QT nanoparticles reversed cognition and memory impairments when injected into APP/PS1 mice insulted by AD. Furthermore, the improvement of spatial memory was also observed in a dose-dependent manner. The observations made make quercetin encapsulated in PLGA a potent inhibitor against Aβ42 aggregation and cytotoxicity with improved therapeutic efficacy for AD treatment.

### 5.5. Liposomes

Another example of widely used nanosized carriers for targeted drug delivery are liposomes, phospholipid spherical vesicles consisting of at least one concentric lipid bilayer that mimics the natural biomembrane that encloses aqueous spaces. Liposomes, together with solid lipid nanoparticles, are representative of lipid-based nanoparticles. Their sizes range from 30 nm to several micrometers, but the optimum size to pass through the BBB and provide efficient drug delivery is 20–100 nm. All lipophilic, hydrophilic, and amphiphilic molecules can be encapsulated, transferred, released, and protected from early inactivation and degradation [163]. Priprem et al. developed intranasal quercetin liposomes and tested them in male Wistar rats [164]. The elevated plus maze test showed anxiolytic-enhancing effects of quercetin liposomes, while the Morris water maze test revealed cognitive function improvement in rats compared with free quercetin treatment animals. It is worth emphasizing that administration through the intranasal route led to the direct delivery of quercetin to the brain, bypassing the BBB. The intranasal path provides more efficient delivery to the brain of lower quercetin doses and a lack of cytotoxicity compared to oral dosage.

α-Mangostin (α-M) is another polyphenolic compound that, apart from numerous pharmacological effects, slows down the progression of Alzheimer’s disease by affecting the amyloidogenic process [165]. Its neuroprotective effect is limited because of its poor penetration through the blood-brain barrier. In the study by Chen et al., liposome modification by transferrin was applied, thus the penetration of the drug across the BBB was facilitated via receptor-mediated endocytosis [166]. Such an application was used to examine α-mangostin transport in the Tf(α-M) liposome through the BBB and its distribution in the rats’ brains. The transport of the Tf(α-M) liposome through the BBB was confirmed by researchers in an in vitro BBB model constructed of cocultured astrocytes and the bEnd3 cells. α-M accumulation in the brain was confirmed in in vivo experiments on rats’ brains after intravenous administration. The authors showed the improvement of the brain delivery of α-mangostin by transferrin-modified liposome constructs and proved that Tf liposomes can serve as a potential carrier of the drug against AD.

## 6. Plausible Adverse Effects of Polyphenol Supplementation

Polyphenols, as biologically active substances, can be at risk of inducing some adverse events in response to their ingestion. Increasing doses, long-term treatment, accumulation of these compounds with elevated plasma concentrations due to renal or liver insufficiency, and interactions with other drugs may increase the risk of the occurrence of side effects [167,168]. In general, the mechanism of polyphenol toxicity may be related to pro-oxidant activity, genotoxicity, effect on endocrine systems, inhibition of iron absorption, and changes in the pharmacokinetics of other medications (Table 5).

To reduce the risk of this toxicity related to polyphenol ingestion, it is necessary to establish the maximum safe daily dose and maximum plasma concentration of the provided polyphenol and its main metabolites. Moreover, analysis of plausible interactions of phenolics with concomitant medications as well as comorbidity in individual subjects should be always taken into account before the start of treatment with polyphenols.

## 7. Factors That May Influence the Efficacy of Polyphenols in the Prevention of Neurodegenerative Diseases

Polyphenols can act as neuroprotective agents through indirect and direct actions. Indirect action involves all beneficial processes occurring outside the brain, while direct action involves traveling through the blood-brain barrier into brain tissue. Therefore, indirect action, the area under the plasma concentration time curve (AUC) as a measure of the overall exposure of tissues to ingested phytochemicals, is crucial [179]. The plasma concentration of ingested polyphenol as well as the AUC are the net results of polyphenol’s bioavailability, metabolism, and excretion in urine and feces [180]. Polyphenol’s bioavailability differs greatly from one compound to another and is determined by solubility, the degree of polymerization, conjugation, or glycosylation resulting from the chemical structure [168]. In general, ingested polyphenols undergo intestinal transformation by enterocytes and gut microbiota into fewer complex compounds, which, after absorption, enter the liver via portal circulation, where additional enzyme-catalyzed modifications take place. Subsequently, with the stream of mixed venous blood, they reach the heart, are distributed to other organs and tissues, including the kidneys, and are excreted with the urine [180]. The issue of polyphenol bioavailability was reviewed in detail elsewhere [7,179,180]. Therefore, Table 6 briefly summarizes the main factors influencing polyphenol bioavailability. The vast majority of studies on plant polyphenol bioavailability were performed on healthy subjects [7,179,180]. Therefore, any pathological processes in the gastrointestinal tract leading to impaired intestinal absorption, as well as antibiotic treatment resulting in the suppression of gut microbiota, may decrease polyphenol bioavailability. On the other hand, decreased glomerular filtration in the course of renal insufficiency may result in polyphenol accumulation and an increase in plasma levels.

Despite the differences between polyphenol’s classes, their bioavailability is low. These results are due to low polyphenol solubility and their presence as polymers or glycosylated forms [7,180]. There are some technological approaches that may increase polyphenol bioavailability when ingested with food or dietary supplements [181]. This includes food processing to facilitate polyphenols release from food matrix (mechanical—juice, puree) thermal (drying, pasteurization, sterilization-net result depends on amounts of compound released from food matrix and amount of compound degraded by these treatments, this also destroys polyphenol oxidase enzyme degrading polyphenols), nonthermal (high pressure homogenization), oral-delivery nanoformulations (described in the previous section, improves polyphenols stability, overcomes problems with light sensitivity and low solubility in water, and enhances bioavailability in experiments on animals), enzymatic hydrolysis (e.g., by β-glucosidase) into more bioavailable compounds (increases solubility, conversion by enterocyte and microbial enzymes), fermentation (bacterial enzymes convert polyphenols to less complex compounds with higher bioavailability), and coadministration with probiotics (e.g., lactobacillus species, probiotic strains induce direct polyphenols hydrolysis and modify the gut microbiota thus enhance bioavailability) [181]. However, it should be pointed out that these technological and biotechnological treatments are not successful for all products rich in polyphenols and, in some cases, are without beneficial effect or even decrease polyphenol bioavailability [181]. On the other hand, changes in eating habits that consume more products rich in plant polyphenols increase the ingested daily polyphenol dose, and thus, even in the case of the same bioavailability, increase the polyphenols AUC and probably their neuroprotective effect. Low-molecular-weight phenolics produced from ingested polyphenols by the gut microbiota seem important for direct neuroprotection [179]. They have higher bioavailability [179] and cross blood-brain barriers (e.g., caffeic acid) [8,68,182]. Moreover, these compounds can be metabolized by endothelial cells to generate numerous derivatives with various biological activities [8]. Therefore, biotechnological food treatments (enzymatic hydrolysis, fermentation, coadministration with probiotics) that raise the plasma concentrations of these low-molecular-weight phenolics can facilitate their transportation into brain tissue and increase the neuroprotective effect. Another promising approach could be dietary supplementation with nanocapsules containing these active metabolites (postbiotic metabolites) [181]. It is also possible to pharmacologically stimulate phenolic transportation through blood-brain barriers; however, this requires further studies [8,182].

## 8. Conclusions and Perspectives

Numerous clinical and observational studies showed a protective effect of a diet rich in plant polyphenols or diet supplementation with polyphenol-rich extracts against neurodegenerative processes in humans. Although the mechanism of action of these phytochemicals is complex (involving indirect and direct effects) and not fully understood, one may conclude based on the aforementioned studies that increased dietary consumption of plant polyphenols is beneficial for the CNS.

However, in the case of a single polyphenol and a defined mixture of isolated polyphenols, the following issues should be solved in future clinical trials:

1. Indications and possible contraindications
2. Optimal composition of the polyphenol mixture
3. Dose and duration of treatment
4. Optimal bioavailability of polyphenols
5. Nanoformulation to increase phytochemical bioavailability and delivery to the CNS, including transportation through the BBB
6. Effectiveness of preparations composed of postbiotic polyphenol metabolites or polyphenols pretreated with hydrolytic enzymes
7. Evaluation of possible side effects
8. Pharmacokinetics in human blood and brain tissue—dependence between plasma concentrations and concentrations in brain tissue or cerebrospinal fluid.

Thus far, only natural polyphenols have been studied as neuroprotectors. Perhaps some modification of the chemical structure of a given polyphenol may increase its neuroprotective activity and transportation through the BBB. Another approach is to pharmacologically modify BBB permeability and stimulate the transportation of polyphenols into the central nervous system. Therefore, numerous questions, including those listed above, should be answered before developing neuroprotective medications based on plant polyphenols.

## Figures and Tables

**Table 1:** Animal studies on polyphenols’ impact on the central nervous system activity and cognitive functions.

Objectives	Study Description	Main Results	References
To assess the impact of **blueberry flavonoids** on long-term memory function.	Male Lister-hooded rats were divided into three groups—young group approximately 6 months old, aged group approximately 18 months old and aged group approximately 18 months old blueberry-supplemented. Supplementation with the blueberry diet (2% *w*/*w*) was conducted for 12 weeks. Rats were tested in a cross-maze apparatus. Hippocampal and cortical regions were analyzed.	Blueberry supplementation results in improvement in spatial working memory tasks in aged animals. This correlated with the activation of CREB and increased BDNF levels in hippocampus. Additionally, increased phosphorylation level of ERK1/2 was observed.	[33]
To assess changes in hippocampal plasticity parameters (hippocampal neurogenesis, extracellular receptor kinase activation, IGF-1 and IGF-1R levels) after **blueberry supplementation**.	19-months old male F344 rats were divided into two groups—control diet and blueberry extract diet. The dose of blueberry extract was 20 g/kg. Rats were fed with those diets for 8 weeks. Animals were tested in radial arm water maze (RAWM) test.	All tested parameters of hippocampal neuronal plasticity were elevated after blueberry extract supplementation. Observed changes in those parameters correlated with improvements in spatial memory.	[29]
To assess neuroprotective effect of **quercetin** in rotenone-induced hemiparkinsonian rats.	Adult Sprague-Dawley rats were infused with rotenone into the substantia nigra. Quercetin (in doses 25, 50 or 75 mg/kg) was administered at 12 h intervals for 4 days. Mitochondrial dysfunction, oxidative stress, programmed cell death and dopamine neuronal demise were assessed.	Quercetin up-regulates electron transport chain (ETC) complex-I activity in damaged or normal dopaminergic neurons. It also effectively scavenge OH generated in the mitochondria, and completely reverse striatal dopamine loss and nigral glutathione depletion. Quercetin has also blocked programmed cell death in nigral neurons. The effect of quercetin was dose-dependent.	[30]
To assess the effects of **green tea polyphenols** (GTPs) on the permeability of blood-brain barrier (BBB) and expression of caveolin-1 and ERK1/2 after cerebral ischemia.	Cerebral ischemia was induced in rats by middle cerebral artery occlusion (MCAO). Animals were divided into control and GTP group, in each four time points were included: MCA occluded for 0 h, 1 h, 2 h and 4 h. The infarct volume and neurological deficits were assessed. The BBB permeability, caveolin-1 and ERK1/2 levels were determined.	GTPs significantly reduced infarct volume, ameliorated the neurological deficit, and reduced the permeability of BBB. Both caveolin-1 and phosphorylated ERK1/2 expression levels were reduced by GTPs.	[39]
To investigate the effect of Neumentix (containing, among others, **rosmarinic acid**) on inflammation and BBB disruption in transient MCAO (tMCAO).	Mice were treated with vehicle or Neumentix (134 mg/kg/d) for 14 days. Then animals were subjected to tMCAO for 1 h. After this procedure mice kept receiving vehicle or Neumentix for next 5 days.	Neumentix reduced infarct volume, inhibited expression of Iba-1, TNF-α, MCP-1 and improve the integrity of BBB. Moreover, it ameliorated neurobehavioral impairment observed in the corner test.	[40]
To assess protective effect of **lychee seed polyphenols** (LSP) on BBB integrity.	bEnd.3 cell cultures and APP/PS1 mice were utilized. Expression of tight junction proteins (TJs), activation of Aβ(25–35)-induced NLRP3 inflammasome, permeability of bEnd.3 monolayer and cognitive function of mice were examined.	LSP protects BBB integrity through inhibiting Aβ(25–35)-induced NLRP3 inflammasome activation via the AMPK/mTOR/ULK1-mediated autophagy. LSP significantly reduced the permeability of bEnd.3 monolayer. It also improved the spatial learning and memory function in tested mice and increased the expression of TJs.	[41]
To investigate the effect of **resveratrol** on stress-related depression, anxiety, intestinal and visceral dysfunction in rat model of irritable bowel syndrome (IBS).	Rats received chronic acute combining stress (CACS) for 22 days. Resveratrol was administered at doses of 10, 20 and 40 mg/kg 50 min before CACS procedure. Changes in behavior, visceral sensitivity and intestinal motility were measured by the forced swimming, marble bury, abdominal withdrawal reflex (AWR) and intestinal tract motility (ITM) tests.	Resveratrol dose-dependently normalized CACS-induced dysfunctions. This polyphenol alleviate IBS-like effects on depression, anxiety, visceral hypersensitivity and intestinal motility abnormality through regulation of 5-HT1A-dependent PKA-CREB-BDNF signaling in the brain-gut axis.	[42]
To investigate the therapeutic potential of **resveratrol** on sevoflurane-induced cognitive impairment.	6 day-old mice received anesthesia with 3% sevoflurane 2 h daily on postnatal days (P) 6, P7 and P8. Resveratrol (100 mg/kg) was administered for 6 consecutive days to neonatal mice before anesthesia. Animals were tested on mouse Morris water maze (MWM) test.	Resveratrol reversed the effect of repeated sevoflurane exposure and modulated SIRT1-NF-κB pathway in microglia leading to amelioration of cognitive impairment in neonatal mice.	[38]
To assess the potential of **resveratrol** to ameliorate ischemia-induced neuronal cell death.	Mongolian gerbils were divided into three groups: sham control, ischemia and ischemia treated with resveratrol. Transient global cerebral ischemia was induced for 5 min. Resveratrol was injected (30 mg/kg), either during or shortly after occlusion, and again 24 h after ischemia.	Resveratrol significantly decreased delayed neuronal cell death (DND) and glial cell activation. The analysis of resveratrol’s bioavailability indicated that this compound can cross the blood-brain barrier.	[37]
To investigate neuroprotective potential of **caffeic acid (CA)**.	Mice were subjected to a permanent middle cerebral artery occlusion. Mice were pretreated and post-treated with CA (2, 20, 60 mg/kg) at 24, 48, 72, 96 or 120 h after ischemia. Animals were evaluated for brain infarction, neurological deficit score, locomotor activity, working memory, short-term aversive memory, long-term aversive memory and spatial memory.	CA reduced the infarcted area and improved neurological deficit scores. CA also alleviate working, spatial and long-term aversive memory deficits. It also inhibited the ischemia-induced reduction in the synaptophysin level.	[43]

**Table 3:** Impact of polyphenols in the diet on cognitive brain functions.

Study Objective	Study Group	Study Description	Main Results	Reference
Determination of the effect of a blueberry smoothie containing 253 g of anthocyanins on particular aspects of executive functions	Randomized, single-blind, parallel-group study, 54 healthy schoolchildren (aged 7–10)	Study group received a 200 mL WBB drink (253 mg anthocyanins) or a matched placebo. Verbal memory (AVLT), executive function (MANT), and reading efficiency (TOWRE-2) were evaluated before and after 2 h of consumption	Consumption of blueberries significantly improved the memory and attentional aspects without affecting reading efficiency	[62]
Determination of the acute effect of a smoothie of berries on the improvement of executive functions and mood	Single-blind, randomized, placebo-controlled, between-subjects study, 40 healthy subjects (aged 20–30)	The study group received a smoothie of berries (blueberries, raspberries, and strawberries) containing 14.3 g of polyphenols or placebo. Executive function (MANT, switching tasks) and mood (positive and negative affect schedule) were assessed at baseline and 2, 4, and 6 h after consumption	Smoothie consumption maintained cognitive performance during the working day compared to placebo, while not influencing the mood	[63]
Determination of the influence of polyphenol-rich grape and blueberry extract (PEGB) on memory	A bicentric double-blind, randomized, placebo-controlled clinical study, 215 healthy elderly people (aged 60–70 years, BMI 20–30, 26 < MMSE score ≤ 29)	The study group received 600 mg/day of PEGB or placebo for 6 months. The Cambridge neuropsychological test automated battery (CANTAB) was assessed at baseline and after 24 weeks of supplementation	Supplementation with PEGB had no effect on memory performance in the whole cohort. However, PEGB supplementation significantly enhanced cognitive performance in elderly people with significant cognitive impairment	[17]
Dietary flavonoids have been studied for cognitive performance	Cohort study: 2044 adult men and women from southern Italy	The demographics and dietary habits of 808 adults living in southern Italy were analyzed. Food frequency questionnaires (FFQs) were used to assess dietary intake. Data on the polyphenol content of foods were estimated using the Phenol-Explorer database. The Short Portable Mental Status Questionnaire was used as a screening tool for cognitive status	Diet rich in flavonoids negatively correlates with cognitive dysfunction in the adult population. It has been shown that anthocyanins, flavan-3-ols, flavonols, and catechins significantly improve cognitive performance	[64]

AVLT—auditory-verbal learning test, BMI—body mass index, CANTAB—the Cambridge neuropsychological test automated battery, MANT—modified attention network task, MMSE—mini-mental state examination, PEGB—polyphenol-rich grape and blueberry extract, TOWRE-2—Test of Word Reading Efficiency: Second Edition, WBB—flavonoid-rich wild blueberry.

**Table 4:** Human blood-brain barrier penetration by polyphenols.

Sample	Study Group	Analyzed Polyphenols	Polyphenols Detected in CNS	References
CSF *, plasma *	Twenty-eight patients (age 46 ± 16 years; 18 women and 10 men) with neurological diseases (multiple sclerosis, polyneuropathy, mononeuropathy, meningitis, brain tumor, epilepsy, amyotrophic lateral sclerosis, an Friedreich’s ataxia) on a “western” diet	vanillic acid dihydrocaffeic acid caffeic acid homovanillic acid 3-hydroxyphenyl acetic acid hippuric acid 3-hydroxyhippuric 4-hydroxyhippuric 3,4-dihydroxybenzoic acid chlorogenic acid ellagic acid urolithin A	caffeic acid homovanilic acid 3-hydroxyphenylacetic acid	[68]
CNS *	Six patients (aged 34–61, 3 women and 3 men) with suspicion of multiple sclerosis acute ingestion of green tea	(2)-epigallocatechin glucuronide (2)-epicatechin glucuronide (2)-epicatechin sulfate methyl-(2)-epicatechin sulfate	undetectable in the CSF	[120]
Brain MRI * CSF *	One hundred and nineteen patients with mild-to-moderate Alzheimer’s disease 52-week supplementation with resveratrol (500 mg/day, with a dose increase of 500 mg every 13 weeks, final dose 2000 mg/day)	resveratrol 3-O-glucuronidated-resveratrol (3GRES) 4-O-glucuronidated-resveratrol (4GRES) 3-sulfated-resveratrol (SRES)	resveratrol 3-O-glucuronidated-resveratrol (3GRES) 4-O-glucuronidated-resveratrol (4GRES) 3-sulfated-resveratrol (SRES)	[121]
CSF *	Thirty-six patients with mild-to-moderate Alzheimer’s disease oral curcumin supplementation at a dose of 2 g/day, 4 g/day, or placebo for 24 weeks, then for the next 24 weeks	tetrahydrocurcumin glucuronidated metabolites of curcumin	undetectable in the CSF	[122]
human brain tissue #		quercetin-3-O-glucuronide (Q3GA)	quercetin-3-O-glucuronide (Q3GA)	[123]

# postmortem sample; * samples taken while alive.

**Table 5:** Plausible mechanism of plant polyphenols toxicity.

Mechanism	Predisposing Factors	Example	Reference
Pro-oxidant activity	High polyphenol concentration, presence of iron and H_2_O_2_, and a high pH, auto-oxidation-induced generation of ROS	Epigallocatechin gallate	[169,170,171]
Genotoxicity	High concentration and long-term consumption of high doses	Caffeic acid	[172]
Interaction with pharmaceuticals	Changes in bioavailability and pharmacokinetics of benzodiazepines by inhibition of cytochrome P450 3A4 (CYP3A4)	Naringenin (grapefruit juice)	[173]
Antinutritional effect: inhibition of iron absorption	Long-term consumption of large amounts of tea and coffee	Tannins	[174,175,176]
Interaction with the endocrine system	Large consumption of isoflavones with estrogenic activity	Genistein, daidzein, and glycitein	[177,178]

**Table 6:** Main determinants of polyphenols bioavailability.

Factor	Main Mechanism
Class of polyphenols	The bioavailability is determined by the class of polyphenols and ranks as follows: phenolic acids > isoflavones > flavonols > catechins > flavanones > proanthocyanidins > anthocyanins.
Differences in chemical structure within the polyphenol class	Changes in physicochemical properties: solubility, ability to polymerize, and ability to enter reactions of conjugation.
Small intestine	Hydrolysis of most glycosides—aglycone absorbed by enterocytes, conjugation reactions, methylation, glucuronidation, and sulfation.
Liver	Aglycone-conjugation reactions: methylation, glucuronidation, and sulfation; part of the products goes back to the intestine with bile; part is excreted with urine; the majority enter other organs and tissues through the bloodstream.
Colon	Metabolism by bacterial enzymes to fewer complex compounds, absorption by colonocytes, and part of the ingested polyphenol dose are excreted with feces.
Food matrix	Can protect polyphenols from degradation. The food matrix changes caused by diet modification can affect gut microbiota and improve or decrease bioavailability depending on their composition.

## Data Availability

No new data were created or analyzed in this study. Data sharing is not applicable to this article.

## Abbreviations

6-OHDA	6-hydroxydopamine
Aβ	beta-amyloid
ABC	ATP-binding cassette
AD	Alzheimer’s disease
ADHD	attention deficit hyperactivity disorder
AlCl_3_	aluminium chloride
ALM	amyotrophic lateral sclerosis
α-M	α-mangostin
APP	amyloid precursor protein
APP/PS1	double transgenic mice expressing a chimeric mouse/human amyloid precursor protein and a mutant human presenilin 1
AUC	the area under the plasma concentration time curve
AVLT	auditory verbal learning test
AWR	abdominal withdrawal reflex
BAX	Bcl-2-associated X protein
BBB	blood-brain barrier
BCRP	breast cancer resistance protein
BCL2	B-cell lymphoma 2
BDNF	brain-derived neurotrophic factor
bEnd.3	brain endothelium cell line, cells isolated from brain tissue derived from a mouse with endothelioma
BMI	body mass index
CA	caffeic acid
CANTAB	the Cambridge neuropsychological test automated battery
CAT	catalase
CACS	chronic acute combining stress
CF	cocoa flavanol
CNS	central nervous system
CREB	cAMP response element-binding protein
CSF	cerebrospinal fluid
C-SLNs	curcumin-loaded solid lipid nanoparticles
DND	delayed neuronal cell death
EGCG	epigallocatechin-3-gallate
ERK ½	extracellular signal-regulated kinase ½
ETC	electron-transported chain
FDA	the Food and Drug Administration
GMO	glyceryl monooleate
GPX1	glutathione peroxidase 1
3GRES	3-O-glucuronidated-resveratrol
4GRES	4-O-glucuronidated-resveratrol
GSH	glutathione
HD	Huntington’s disease
HPLC-MS2	High-performance liquid chromatography-tandem mass spectrometry
5-HT	5-hydroxytryptamine
6-OHDA	6-hydroxydopamine
IBS	irritable bowel syndrome
ITM	intestinal tract motility
JNK	c-Jun N-terminal kinase
LPS	lipopolysaccharide
LSP	lychee seed polyphenols
MANT	modified attention network task
MAO	monoamine oxidase
MCAO	middle cerebral artery occlusion
MCP-1	monocyte chemoattractant protein-1
MMSE	mini-mental state examination
MRP2	multidrug-resistant protein type 2
MWM	Moris water maze
NADPH	nicotinamide adenine dinucleotide phosphate
NanoCurc	nanoparticle-encapsulated curcumin
NFkB	nuclear factor kappa-light chain enhancer of activated B cells
NSC	neural stem cells
NO	nitric oxide
OX26	antitransferrin receptor antibody
PACA	poly (alkyl cyanoacrylate)
PAL	paired associate learning
PD	Parkinson’s disease
PEGB	polyphenol-rich grape and blueberry extract
PGA	poly (glycolic acid)
P-gp	P-glycoprotein
PLA	poly (lactic acid)
PLGA	poly (D, L-lactide-co-glycolic acid)
PLGA@QT	PLGA-functionalized quercetin nanoparticles
PSA	polysaccharide A
QNPs	quercetin nanoparticles
QT-SPIONs	quercetin-conjugated superparamagnetic iron oxide nanoparticles
RAWM	radial arm water maze
RCTs	randomized control trials
ROS	reactive oxygen species
SCFA	short-chain fatty acids
SH-SY5Y	a thrice-subcloned cell line derived from the SK-N-SH neuroblastoma cell line. It serves as a model for neurodegenerative disorders
SIRT1	Sirtuin 1
SK-N-SH	neuroblastoma cell line
SLNs	solid lipid nanoparticles
SOD1	superoxide dismutase 1
SRES	3-sulfated-resveratrol
Tet-1	ten-eleven translocation methylcytosine dioxygenase 1
Tf(α-M)	α-M liposomes modified with transferrin
TfR	transferrin receptor
TLR4/NFkB	toll-like receptor 4/nuclear factor kappa B
TNF-alpha	tumor necrosis factor–alpha
TOWRE-2	Test of Word Reading Efficiency—second edition
WBB	flavonoid rich wild blueberry
